# Impact of the COVID-19 Pandemic on Healthcare Activity in the Regional Hospitals of Andalusia (Spain)

**DOI:** 10.3390/jcm11020363

**Published:** 2022-01-12

**Authors:** Antonio Lopez-Villegas, Rafael Jesus Bautista-Mesa, Miguel Angel Baena-Lopez, Antonio Garzon-Miralles, Miguel Angel Castellano-Ortega, Cesar Leal-Costa, Salvador Peiro

**Affiliations:** 1 Social Involvement of Critical and Emergency Medicine, CTS-609 Research Group, Poniente Hospital, 04700 El Ejido, Spain; antoniolopezvillegas@andaluciajunta.es; 2 Economic-Financial Subdirection, Alto Guadalquivir Health Agency, 23740 Andujar, Spain; rafael.bautista.mesa@gmail.com; 3 Medical Subdirection, Poniente Hospital, 04700 El Ejido, Spain; miguelangel.baena@ephpo.es; 4 Computer Systems and Communications Area, Poniente Hospital, 04700 El Ejido, Spain; antonio.garzon@ephpo.es; 5 Medical Subdirection, Alto Guadalquivir Health Agency, 23740 Andujar, Spain; macastellano@ephag.es; 6 Nursing Department, University of Murcia, 30120 El Palmar, Spain; 7 Health Services Research Unit, Foundation for the Promotion of Health and Biomedical Research of Valencia Region (FISABIO), 46020 Valencia, Spain; peiro_bor@gva.es

**Keywords:** COVID-19, healthcare activity, regional hospitals, Spain, telemedicine

## Abstract

(1) Background: The large global outbreak of severe acute respiratory syndrome coronavirus 2 (SARS-CoV-2) has overloaded the public health systems and reduced the regular healthcare activity, leading to a major health crisis. The main objective of this study was to carry out a comparative evaluation of the healthcare activities in the hospitals of Eastern Andalusia, Spain. (2) Methods: In this study, an observational, multicentered, and retrospective approach was adopted to compare the healthcare activities of the Poniente Hospital (PH) and the Alto Guadalquivir Health Agency (AGHA). Data was collected over a period of 24 months, i.e., from 1 January 2019 to 31 December 2020, and the variables evaluated were: patients seen in the hospital emergency service (HES), X-ray tests performed, patients cited in outpatient consultations, surgical interventions performed, and patients included in the waiting list. (3) Results: The analysis of the above-mentioned variables revealed a significant reduction in the number of patients registered in 2020 at HES as compared to that in 2019 for both PH (*p* = 0.002) and AGHA (*p* < 0.001). Moreover, the number of surgical interventions in 2020 was significantly reduced from that in 2019 for both PH (*p* = 0.001) and AGHA (*p* = 0.009). Moreover, for PH (*p* < 0.001), a significant reduction was observed in the waiting list admissions in 2020 compared to that in 2019; however, no significant difference in the waiting list admissions between the years 2020 and 2019 was observed for AGHA (*p* = 0.446). In 2020, the number of teleconsultations was significantly increased from that in 2019 for both PH (*p* < 0.001) and AGHA (*p* = 0.006). (4) Conclusion: The analysis carried out indicates that in 2020, compared to 2019, healthcare activity was significantly reduced in most of the parameters included in this study.

## 1. Introduction

Since the Chinese health authorities notified the WHO of the first case of a type of pneumonia (Wuhan on 31 December 2019), the lives of millions of people around the world have significantly changed [1,2,3]. In a matter of days, the severe acute respiratory syndrome coronavirus 2 (SARS-CoV-2) rapidly spread around the world, leading to the first pandemic of the 21st century [4,5,6]. To date, 281,808,759 million people have been infected with SARS-CoV-2 worldwide, leading to 5,411,744 deaths [7].

### Impact of the COVID-19 Pandemic in Spain through 2020

Since the reporting of the first case of COVID-19 in Spain (La Gomera, Canary Islands) at the end of January 2020 [8], there has been a steady increase in the number of people infected (6,667,511) [9] and the number of deaths (89,573) [10,11].

However, data provided by the Spanish National Institute of Statistics and published in different media [12], confirm that in 2020 the pandemic caused more than 80,000 deaths in Spain. This represents a difference of 30,081 deaths with respect to the official balance of the Ministry of Health, which on 28 December 2020 stood at 50,122 confirmed deaths due to coronavirus. According to the ENE-COVID seroprevalence study, 1 in 10 Spaniards had been infected by the virus in November 2020. [13]. The global health crisis caused by SARS-CoV-2 led to extreme overloading of different public health systems worldwide [14]; standing out among them is the Spanish health system [15,16,17].

In view of the rapid spread of the virus and in order to prevent the spread of the virus, not oversaturating the health system, and being able to focus on providing the best possible health care to patients affected by COVID-19 [6,18], on 14 March 2020, the Spanish Government decreed a state of alarm throughout the national territory, limiting the free movement of citizens to essential acts such as the purchase of food and medicines, going to health centers or the workplace and confining the population to their homes [16,17,18,19]. On 28 March 2020, all non-essential on-site work activities were suspended for 15 days. On 2 April 2020, the highest number of deaths due to coronavirus in 1 day (950) was recorded. As of 28 April 2020, the asymmetric de-escalation plan began nationwide [20].

The saturation of the healthcare system, which was on the verge of collapse in March, April, and May 2020, implied a rapid restructuring of the care processes, as well as a relocation of physical and material resources [6,21,22]. In mid-June, Spain was the fifth country in number of confirmed cases, behind the United States, Brazil, Russia, and the United Kingdom, and the sixth country in number of deaths, behind the United States, Brazil, the United Kingdom, Italy, and France [23].

On 21 June, after 98 days, the state of alarm expired and Spain entered the so-called “new normality”. In the summer of 2020, there were multiple outbreaks in different areas of the country, which degenerated into community transmission. On 21 October 2020, Spain surpassed the one million infected. On 25 October 2020, the Spanish government decreed a second state of alarm to deal with the second wave of infection. Finally, on 27 December 2020, the vaccination campaign began in Spain with the Pfizer and BioNTech vaccine.

The impact of COVID-19 on the care activity of Hospital Emergency Departments (ED) has been studied in large hospitals in Paris (France), Tehran (Iran) and Osaka (Japan) [24,25,26]. However, no studies have been found where an intrahospital comparison has been performed, that is, showing the results of the care activity performed both in the ED and in other departments of the same hospital, comparing the year prior to the pandemic (2019) versus the year of the pandemic (2020). On the other hand, no studies have been found comparing the same parameters of care activity in different regional hospitals of small and medium size (interhospital comparison). Therefore, a study focused on both aspects may be of interest for the management of future outbreaks [6,21,22]. For this reason, the main objective of this study was to evaluate the impact of COVID-19 on care activity in small and medium-sized regional hospitals.

## 2. Materials and Methods

### 2.1. Design

This is a comparative, observational, multicenter and retrospective study designed to analyze healthcare activity in small and medium-sized regional hospitals in the provinces of Almeria (Poniente Hospital -PH-), Cordoba and Jaen (Alto Guadalquivir Health Agency -AGHA-) in both cases belong to Andalusia (Spain). Data collection was performed over a 24-month period, from 1 January 2019 to 31 December 2020. Table 1 shows the main characteristics of both healthcare centers.

### 2.2. Data Collection

The variables included in the analysis were: users who were treated in the hospital emergency service (HES), number of diagnostic tests (X-ray and laboratory tests were not included), number of radiodiagnostic tests, number of laboratory tests, number of patients cited in outpatient consultations, number of cited patients not presented, number of outpatient consultations carried out over the phone, number of surgical interventions, and finally, patients enrolled on a waiting list. Although information on hospital admissions was collected, as they are the main cost driver in terms of resource use, the information obtained was not taken into account for this manuscript as it will be included in a subsequent cost-based article. The activity data were obtained from the economic management departments of both healthcare institutions.

### 2.3. Ethical Considerations

The protocol of this study was approved by the Regional Ethics Committee for Health Research (CEIC-AL: 91/2020). The present study was conducted under the precepts of the Declaration of Helsinki [29] and Spanish laws on data protection and patient rights [30,31].

### 2.4. Statistical Analysis

Firstly, a descriptive analysis was carried out. Quantitative variables were presented as mean (CI), standard deviation, median, and range (minimum and maximum). Shapiro-Wilk test was used to assess the normality of all the numerical variables. The differences between the hospitals were evaluated through a *t*-test within the normality assumption, and the Mann-Whitney U test was performed for all the features without an assumption of normality. To assess the change between the pre-pandemic (2019) and pandemic periods (2020), *t*-test for paired data (normally distributed data) and Wilcoxon test were applied (abnormally distributed data). All of the statistical analyses were carried out with free software R version 4.0.5 (Vienna, Austria, https://www.r-project.org/), and the *p*-value of ≤0.05 was considered statistically significant.

## 3. Results

### 3.1. Healthcare Activity Carried out in Both Healthcare Institutions Pre- and during the First Year of the Pandemic

Hospital emergency services of Poniente Hospital attended fewer visits in 2020 than in 2019 (*p* < 0.01; Table 2). Regarding the diagnostic tests performed, the number of X-rays reduced by 11.56% (*p* = 0.054). On average, in 2020, a total of 4472 fewer patients were cited in the hospital’s outpatient clinics than in 2019, which represents a reduction of 18% (*p* < 0.001). In 2020, the number of cited patients who did not show up for their hospital appointments also reduced by 33% (*p* < 0.001) compared to that of the previous year. The total number of surgeries performed in the year of the pandemic significantly reduced by 31% compared to that of the previous year (*p* < 0.001). The total number of patients on the waiting list was 19% lower (*p* < 0.001) in 2020 than in 2019 (Figure S1).

The analysis of the data collected from the AGHA (Table 2) indicated that throughout 2020 the number of patients treated in the HES reduced by 35% compared to that of 2019 (*p* < 0.001). The number of X-ray tests performed significantly reduced by 16.74% (*p* < 0.05). In the year of the pandemic, 4710.08 fewer patients were referred to outpatient clinics than in 2019, which represents a reduction of 16% (*p* < 0.001). In 2020, the number of cited patients who did not show up for their appointments reduced by 60% (*p* < 0.001) than in 2019. The number of surgical interventions performed in 2020 was significantly reduced by 25% compared to that in 2019 (*p* < 0.01). In 2020, the total number of patients enrolled in the waiting list decreased by 4% compared to that of the pre-pandemic year (*p* = 0.446) (Figure S2).

**Figure 1:**
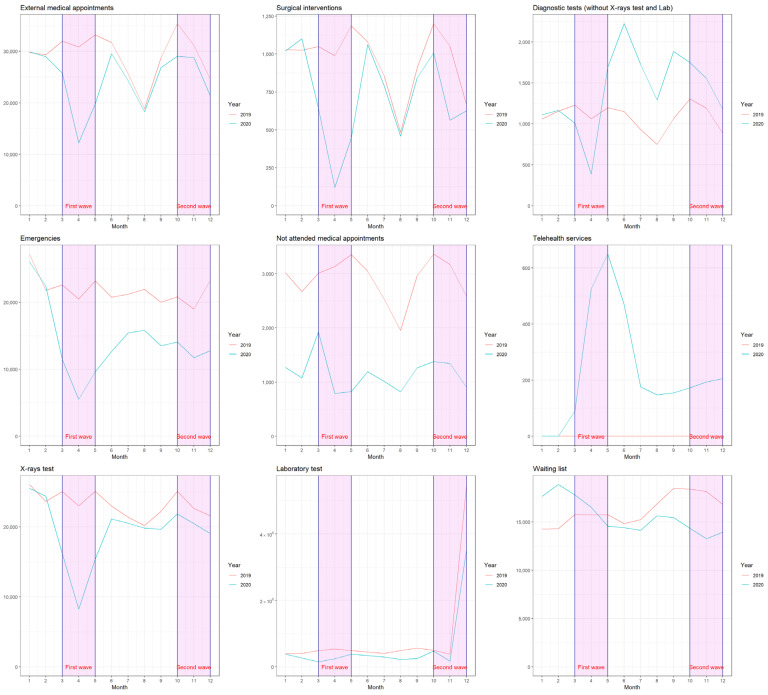
Monthly evolution for the years 2019 and 2020 in the Alto Guadalquivir Health Agency. The first wave and lockdown of Covid-19 in Spain took place between March and May 2020, while the second wave and semi lockdown was between October and December 2020.

In the AGHA, the results for most of the parameters analysed in January 2020 were very similar to those obtained in January of the previous year. However, a very strong fall in the variables was observed in April 2020, coinciding with the first month of strict lockdown (Figure 1).

**Figure 2:**
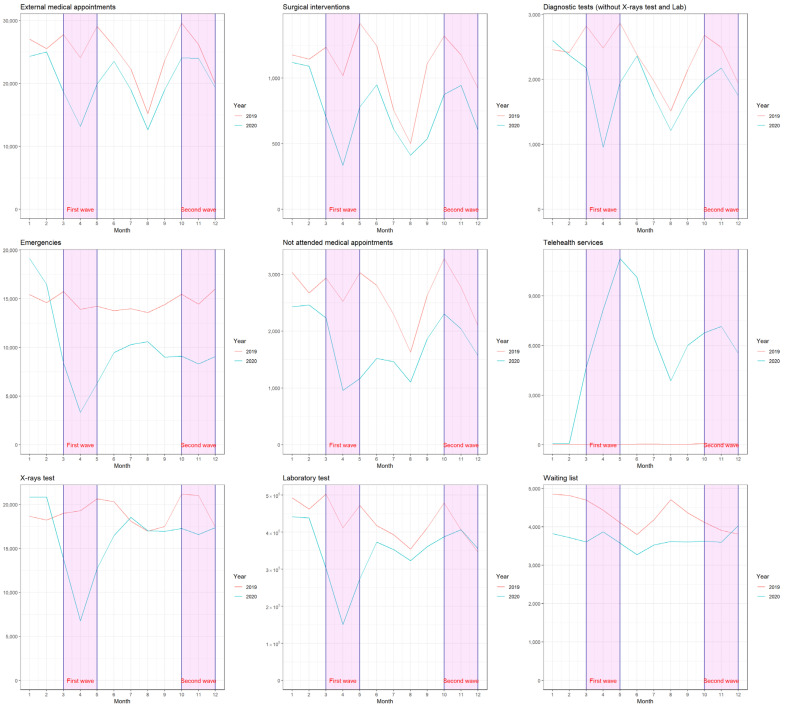
Monthly evolution for the years 2019 and 2020 at the Poniente Hospital. The first wave and lockdown of Covid-19 in Spain took place between March and May 2020, while the second wave and semi lockdown was between October and December 2020.

These results coincide with those achieved by PH (Figure 2); the levels of the variables did not recover throughout 2020, except for X-ray and laboratory tests.

### 3.2. Comparison of Healthcare Activity between PH and AGHA

Table 3 compares the results of the main health activity parameters between PH and AGHA in 2 different years, first in 2019 and then in 2020. With respect to the year 2020, there were significant differences in the number of outpatient consultations cited (*p* = 0.042), number of patients cited and not presented at the hospital (*p* < 0.01), number of diagnostic tests performed (*p* = 0.019), number of patients attended to at the HES (*p* = 0.042), number of outpatient consultations performed via telephone (*p* < 0.01), and the total number of patients included in the waiting list (*p* < 0.001) (Figures S3 and S4).

## 4. Discussion

### 4.1. Main Findings

The results of the comparative analysis carried out in this study quantify the reduction in healthcare activity, which began in February 2020 (in most of the parameters analyzed) and accentuated in the months of March and April 2020 as a result of the declaration of the state of alarm and the subsequent lockdown in Spain. The comparison of the healthcare activity between the years 2020 and 2019 in both the health institutions revealed a significant reduction in the number of outpatient consultations cited, outpatient consultations cited but not presented, number of surgical interventions, number of diagnostic tests, patients seen in the HES, number of X-rays tests, number of laboratory tests, and the total number of patients included in waiting list. Interestingly, the number of outpatient consultations carried out over the telephone significantly increased.

By March 2020, the hospitals and primary care centers were subjected to immense pressure, as several steps were taken to prevent the spread of the virus. Visits were prohibited, and all non-urgent health activities were reduced to a minimum because of a lack of material and human resources to fight the pandemic and at the same time maintain regular health activities [32]. The health emergency caused by the COVID-19 pandemic put the professionalism and organizational structures of healthcare systems to test. Practically overnight, healthcare procedures changed from the traditional way of working to deal with a large number of transformations, some of which were not foreseen and others were long awaited [33].

The analysis of the included variables indicated that, in 2020, the total number of patients who were treated at PH and AGHA reduced by 32% and 35%, respectively, than in the previous year. This reduction coincides with the results presented in previous studies [32,34,35] and may have been caused either due to the fear of being infected or the belief that the patients can heal themselves in their own homes. In a previous study [36], comparing the annual gross rate of visits to HES and admissions between the year 2020 and the average of the previous 4 years, it was observed that the number of visits to HES reduced by 83%. While in another study [37] carried out in a tertiary hospital, the total number of consultations carried out in the HES reduced by 68%, similar to that of another study [38] carried out in a second-level hospital center, wherein the reduction in the number of visits was 64%.

The surgical activity was reduced at both PH (31.22%) and AGHA (24.67%). This decrease is lower than that found in 2 previously published studies [34,38]. In the first study, scheduled activity was reduced both in trauma (86%) and in surgery (92%), whereas in the second study carried out in the pediatric surgery unit of a tertiary hospital, surgical interventions were reduced by 98%. This significant drop in surgical activity was largely due to a management decision anticipating a higher bed occupancy rate, as well as the need to have a higher number of healthcare professionals assisting COVID-19 patients [34].

The number of patients referred to outpatient clinics was reduced both at PH (18%) and AGHA (16%). These results are similar to those found in a previous study [39], based on the number of first consultations (new patients), in which the number of patients cited in outpatient consultations decreased by almost 21%. The number of follow-up consultations made over the phone increased by 100% in PH, while in the case of AGHA, it jumped from 0 to 232 appointments in just one year. These results are once again similar to that of a previous study [15], where the number of follow-up consultations made over the telephone multiplied by a value of 3; also, a one-third decrease in the number of face-to-face consultations was observed in 2020 than in 2019. In addition, as of June, the face-to-face consultations returned to the figures observed in 2019 [15]. However, the results of this study indicated that despite an increase in face-to-face consultations during 2020, the values observed in 2019 were never reached.

Another aspect to be highlighted, which coincides with both the PH and the AGHA, is the lack of growth in the total number of patients on the waiting list. In contrast, the results presented in other studies, such as that of the Spanish Association Against Cancer [40] (SAAC). In the study carried out by the SAAC, there is an increase in the number of patients included in the waiting lists, which may be due to several reasons, such as patients not preferring to visit visiting hospitals out of fear or a health system that has most of its resources rechanneled to ensure the care of COVID-19 patients. This led to significant delays in addressing other health issues during the peak months of the first wave (March to June 2020).

### 4.2. Limitations and Strengths

Analysis of the healthcare activity had some methodological weaknesses and strengths that need to be taken into account. Firstly, although information on hospital admissions was collected, as they are the main cost driver in terms of resource use, the information obtained was not taken into account for this manuscript, as it will be included in a subsequent cost-based article. Secondly, the results of this study provide relevant insights into the consequences of COVID-19 on regular healthcare activities at hospitals. However, any other conclusion about the clinical consequences of these changes in hospital care cannot be drawn from this study. The third limitation is the cost analyses, which refers to the possibility to extrapolate the results of this study to other areas. This limitation is directly related to the particular incidence of COVID-19 in the population of a particular area that was not included in this study. Finally, the fourth limitation is that because of the properties and collection process of the data, proportions between paired data could not be compared (pre-pandemic and pandemic periods). This was because the total size of the sample for each variable was unknown, leading to the assessment of positive outcomes but not the negative ones.

The strengths of this study are largely related to the exhaustive description of the reduction in hospital care activity as a consequence of COVID-19. The results can be utilized by the managers of public healthcare services to further research the clinical impact of COVID-19 in areas not included in this study. In addition, future cost-opportunity studies could reveal the indirect costs borne by patients and public health systems because of the COVID-19-driven reduction in hospital care activities.

## 5. Conclusions

The results of this study demonstrate that the healthcare activity in regional hospitals of Eastern Andalusia (Spain) significantly decreased during 2020, mainly due to the general confinement of people during the peak period of the first wave (March to June) and subsequent months of the year. The findings of this study can be utilized to better meet the challenges of future pandemics.

## Figures and Tables

**Table 1:** Main characteristics of the healthcare institutions included in the study.

	Poniente Hospital [27]	Alto Guadalquivir Health Agency [28]
Reference population (inhabitants)	264,386	264,733
Municipalities covered	15	47
Total professionals working	1419	1874
Outpatient Consultations	280,337	403,490
Emergencies	151,170	255,833
Surgical activity	12,516	11,613
Admissions	14,067	8874
Deliveries	2567	961
Laboratory diagnostic tests	3,966,819	4,638,51
Diagnostic radiology tests	165,680	281,883
Healthcare centers included	(1) Adra: Adra health center and La Curva and Puente del Rio clinics. (2) Berja: Berja Health Center and the clinics of Alcolea, Balanegra, Dalías, Fondón, Láujar de Andarax, Paterna del Rio, Bayárcal and Fuente Victoria. (3) El Ejido: Health Centers of El Ejido, El Ejido Sur, Las Norias and Santa María del Águila and the clinics of Almerimar, Balerma, Matagorda-Guardias Viejas, Pampanico, San Agustín and Santo Domingo. (4) Roquetas de Mar: Aguadulce Sur, El Parador, Las Marinas, Roquetas and Roquetas Sur health centers and the clinics of Aguadulce, Cortijos Marín, Felix, El Solanillo and Enix auxiliary. (5) Vícar: La Gangosa, La Mojonera and Puebla de Vícar health centers and the clinics of Barrio Archilla-Cañada Sebastiana, Cabañuelas Bajas, Llanos de Vícar, Venta del Viso and La Envía.	1. In the province of Jaén: the Alto Guadalquivir Hospital and the High Resolution Hospitals of Sierra de Segura, Alcaudete and Alcalá la Real. 2. In the province of Cordoba: the Montilla Hospital and the Puente Genil and Valle del Guadiato High Resolution Hospitals.

**Table 2:** Main results of the healthcare activity carried out in both healthcare institutions pre- and during the first year of the pandemic.

	Poniente Hospital				Alto Guadalquivir Health Agency			
	2019	2020	Difference	*p*-Value	2019	2020	Difference	*p*-Value
No. of external consultations cited *	24,690.33 ± 4070.60	20,218.17 ± 4189.12	−4472.17 ± 3498.28	<0.001	29,270.42 ± 4433.65	24,560.33 ± 5576.88	−4710.08 ± 5766.78	<0.001
No. of external consultations cited-not presented *	2642.17 ± 456.87	1760.08 ± 535.28	−882.08 ± 471.99	<0.001	2899.67 ± 402.60	1150.17 ± 323.43	−1749.50 ± 420.08	<0.001
No. Surgical Interventions *	1085.75 ± 255.33	746.75 ± 256.05	−339.00 ± 230.92	<0.001	960.08 ± 208.16	723.25 ± 300.49	−236.83 ± 313.50	<0.01
No. Diagnostic tests (not RX or Laboratory) *	2349.92 ± 394.03	1915.25 ± 480.53	−434.67 ± 459.88	<0.01	1081.92 ± 159.64	1414.08 ± 489.00	332.17 ± 485.34	<0.05
No. Emergencies *	14,637.42 ± 822.10	9964.67 ± 4179.52	−4672.75 ± 4046.89	<0.01	21,835.08 ± 2117.57	14,246.17 ± 5447.95	−7588.92 ± 4548.63	<0.001
Number of external telephone inquiries *	40.42 ± 22.26	5850.83 ± 3409.88	5810.42 ± 3404.99	<0.001	0.000 ± 0.000	232.25 ± 206.02	232.25 ± 206.02	<0.01
No. Radiodiagnosis tests *	19,032.33 ± 1467.06	16,261.33 ± 3806.65	−2771.00 ± 4442.76	0.054	23,241.33 ± 1787.42	19,351.50 ± 4520.03	−3889.83 ± 4692.48	<0.05
No. of laboratory tests *	428,910.58 ± 52,088.19	347,075.00 ± 79,617.42	−81,835.58 ± 88,169.37	<0.01	882,306.67 ± 1,451,828.23	558,611.33 ± 943,747.59	−323,695.33 ± 523,559.12	<0.001
No. patients on the waiting list *	4315.42 ± 386.552	3656.33 ± 190.58	−659.083 ± 391.70	<0.001	16,224.58 ± 1522.70	15,557.25 ± 1784.66	−667.33 ± 2921.43	0.446

* Mean ± Standard Deviation.

**Table 3:** Comparison of the parameters included in the study between Poniente Hospital and Alto Guadalquivir Health Agency during 2019 and 2020.

	2019				2020			
	Alto Guadalquivir Health Agency	Poniente Hospital	Total	*p*-Value	Alto Guadalquivir Health Agency	Poniente Hospital	Total	*p*-Value
No. of external consultations cited *	29,270.41 ± 4433.65	24,690.33 ± 4070.60	26,980.38 ± 4774.75	0.015	24,560.33 ± 5576.88	20,218.17 ± 4189.12	22,389.25 ± 5309.06	0.042
No. of external consultations cited-not presented *	2899.67 ± 402.60	2642.17 ± 456.87	2770.92 ± 441.19	0.128	1150.17 ± 323.43	1760.08 ± 535.28	1455.13 ± 533.01	<0.01
No. Surgical Interventions *	960.08 ± 208.16	1085.75 ± 255.33	1022.92 ± 236.69	0.200	723.25 ± 300.49	746.75 ± 256.05	735.00 ± 273.28	0.839
No. Diagnostic tests (not RX or Laboratory) *	1081.92 ± 159.638	2349.92 ± 394.03	1715.92 ± 711.25	<0.001	1414.08 ± 489.00	1915.25 ± 480.53	1664.67 ± 538.81	0.019
No. Emergencies *	21,835.08 ± 2117.57	14,637.42 ± 822.10	18,236.25 ± 3997.81	<0.001	14,246.17 ± 5447.95	9964.67 ± 4179.52	12,105.42 ± 5227.94	0.042
Number of external telephone inquiries *	0.000 ± 0.00	40.42 ± 22.26	20.21 ± 25.75	<0.001	232.25 ± 206.02	5850.83 ± 3409.88	3041.54 ± 3717.05	<0.01
No. Radiodiagnosis tests *	23,241.33 ± 1787.42	19,032.33 ± 1467.06	21,136.83 ± 2679.33	<0.01	19,351.50 ± 4520.03	16,261.33 ± 3806.65	17,806.42 ± 4380.93	0.060
No. of laboratory tests *	882,306.67 ± 1,451,828.23	428,910.58 ± 52,088.19	655,608.63 ± 1,031,021.24	0.198	558,611.33 ± 943,747.59	347,075.00 ± 79,617.43	452,843.17 ± 663,831.72	0.242
No. patients on the waiting list *	16,224.58 ± 1522.70	4315.42 ± 386.55	10,270.00 ± 6178.92	<0.001	15,557.25 ± 1784.66	3656.33 ± 190.58	9606.79 ± 6203.88	<0.001

* Mean ± Standard Deviation.

## Data Availability

The datasets used and/or analyzed in the current study are available from the corresponding author upon request.

## Supplementary Materials

The following supporting information can be downloaded at: https://www.mdpi.com/article/10.3390/jcm11020363/s1. Figure S1: Box diagrams of the variables of interest in the Alto Guadalquivir Health Agency; Figure S2: Box diagrams of the variables of interest in the Poniente Hospital; Figure S3: Box diagrams of the variables of interest, comparison between both hospitals (year 2019); Figure S4. Box diagrams of the variables of interest, comparison between both hospitals (year 2020).
