# Supplementary material for: Impact of the COVID-19 Pandemic on Healthcare Activity in the Regional Hospitals of Andalusia (Spain)
Source: J Clin Med. 2022 Jan 12;11(2):363. doi: 10.3390/jcm11020363 (PMC8781610; doi:10.3390/jcm11020363)
Supplement: Supplementary file 1 [file jcm-11-00363-s001.zip › jcm-1493150-supplementary.pdf]

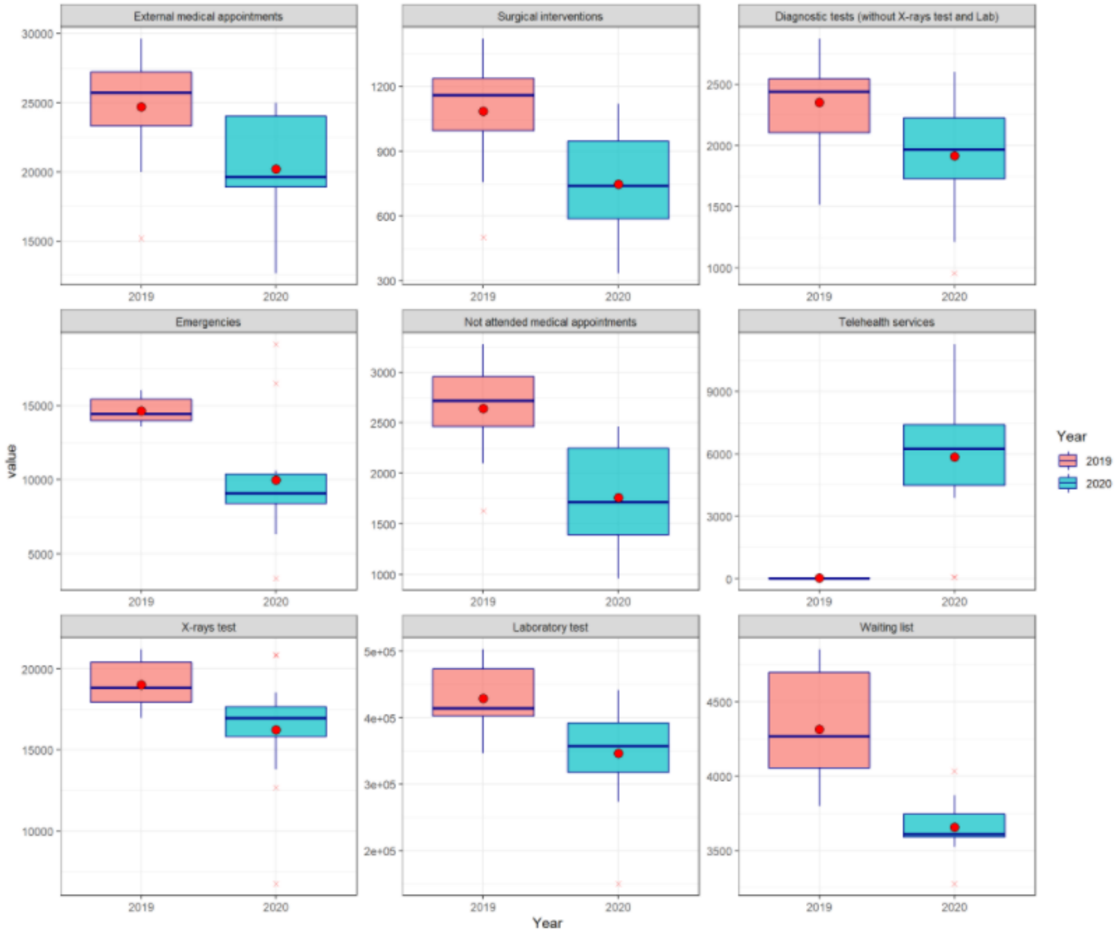

Figure S1. Box diagrams of the variables of interest in the Hospital de Poniente.
* The red point represents the mean value and the blue line the median.

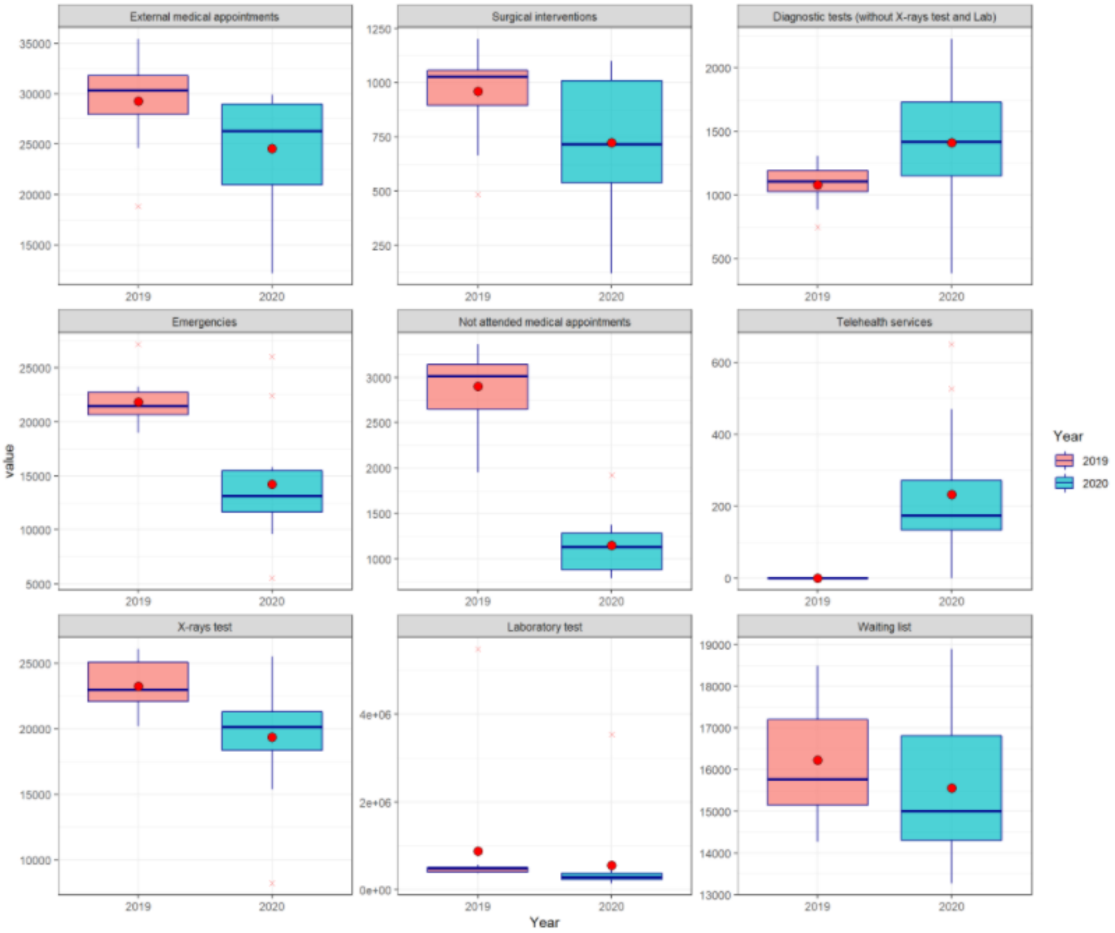

Figure S2. Box diagrams of the variables of interest in the Alto Guadalquivir Health Agency.
* The red point represents the mean value and the blue line the median.

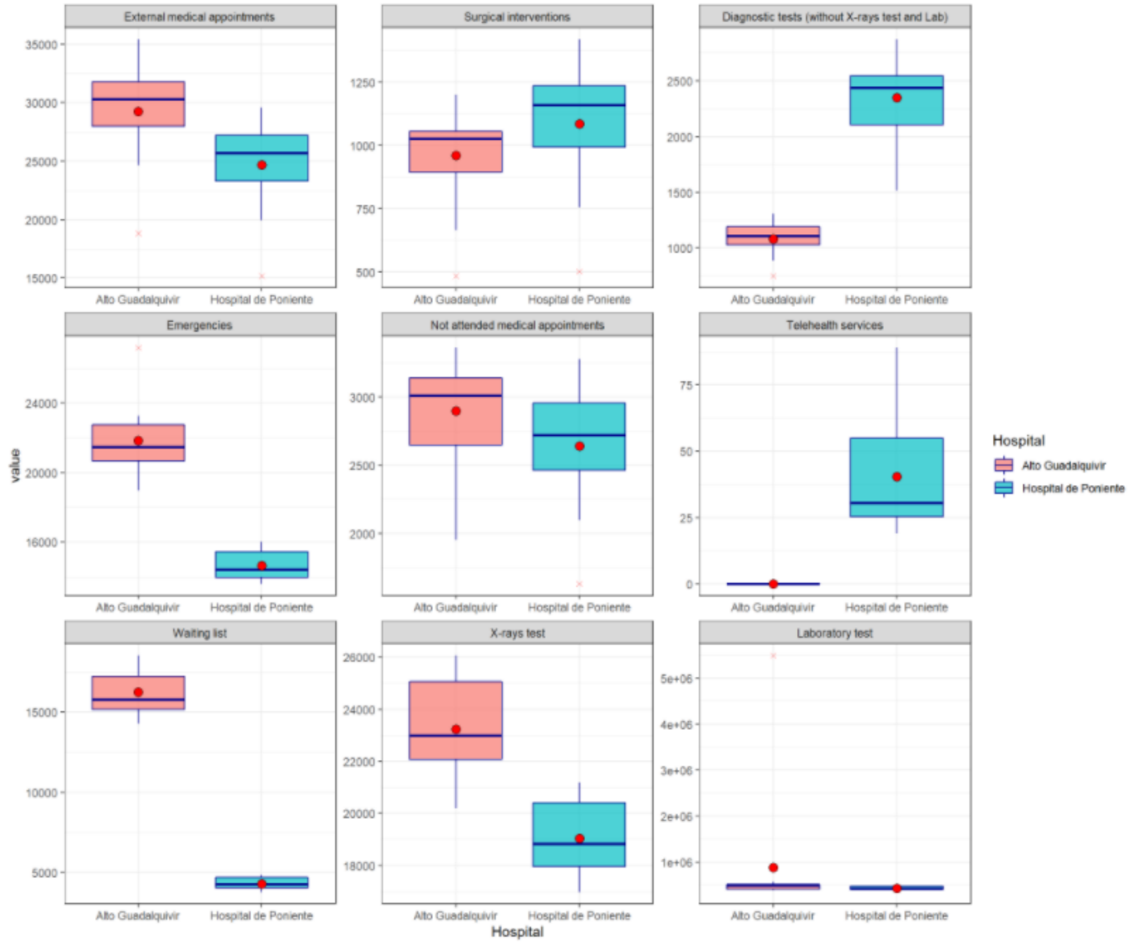

Figure S3. Box diagrams of the variables of interest, comparison between both hospitals (year 2019).
* The red point represents the mean value and the blue line the median.

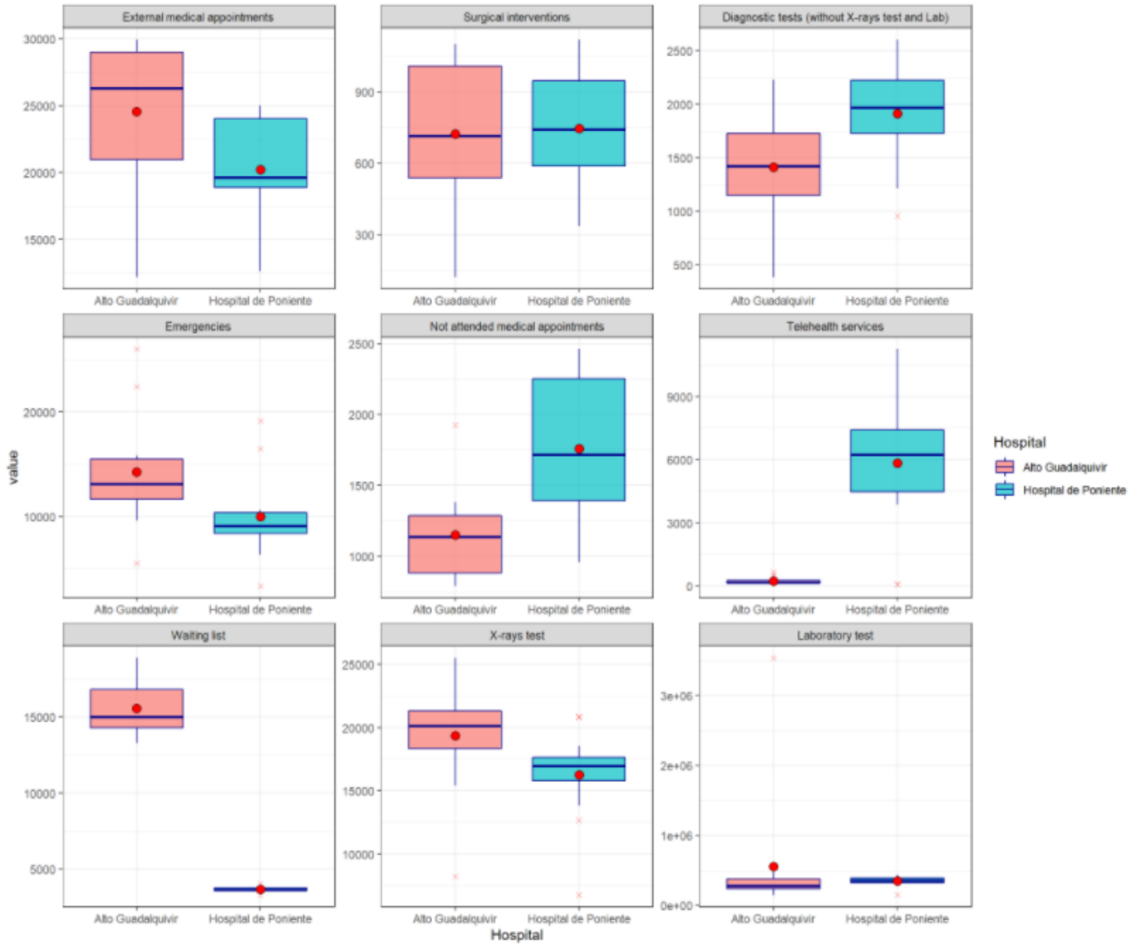

Figure S4. Box diagrams of the variables of interest, comparison between both hospitals (year 2020).
* The red point represents the mean value and the blue line the median.
